# Navigating the path: Advice to physician-scientists on choosing a clinical specialty

**DOI:** 10.7554/eLife.110448

**Published:** 2026-07-03

**Authors:** Talia Swartz, Jose E Cavazos, Marshall Horwitz, Patrick J Hu, Barbara Sampson, Tiffany Scharschmidt, Ali Zarrinpar, Nicholas Mohr, David Mankoff, Jaime Chu, Kyu Y Rhee, Charles W Emala, Christopher S Williams

**Affiliations:** 1 Professor of Medicine, Senior Associate Dean for MD-PhD Education, Icahn School of Medicine at Mount Sinai New York United States; 2 Associate Dean and Professor of Neurology, UT Health San Antonio San Antonio United States; 3 Associate Dean for Physician-Scientist Education, Professor, Department of Laboratory Medicine and Pathology, University of Washington School of Medicine Seattle United States; 4 Associate Dean for Medical Education, Associate Professor of Medicine and Pharmacology, University of Colorado School of Medicine Aurora United States; 5 Cardiovascular Pathologist, Office of Chief Medical Examiner of the City of New York New York United States; 6 Professor, Dermatology, Vice Chair of Research in the Department of Dermatology, UCSF San Francisco United States; 7 Professor of Surgery, Assistant Chair of Innovation, Associate Director of MD-PhD Training Program, University of Florida Gainesville United States; 8 Professor of Emergency Medicine, Anesthesia and Critical Care, and Epidemiology, University of Iowa Carver College of Medicine Iowa City United States; 9 Matthew J. Wilson, Professor of Radiology, Vice-Chair for Research, Department of Radiology, Associate Director of Education and Training, Abramson Cancer Center, University of Pennsylvania Philadelphia United States; 10 Professor of Pediatrics, Endowed Professor of Pediatric Liver Research, the Associate Chief of the Division of Pediatric Hepatology, Icahn School of Medicine at Mount Sinai New York United States; 11 Professor of Medicine and of Microbiology & Immunology, Weill Cornell Medicine New York United States; 12 Henrik H. Bendixen Professor of Anesthesiology, Vice Chair for Research, Department of Anesthesiology, Columbia University New York United States; 13 Associate Dean, Physician Scientist Education and Training, Professor of Medicine, Vanderbilt University Medical Center, Veterans Administration Health System, Vanderbilt Ingram Cancer Center Nashville United States; University of California, Los Angeles United States; University of California, Los Angeles United States

**Keywords:** physcian scientist, specialty selection, career

## Abstract

Choosing a clinical specialty is a critical decision for physician-scientist trainees, influencing both clinical practice and research trajectory. This article provides a structured approach to specialty selection, emphasizing the importance of aligning clinical interests with long-term research goals, evaluating training pathways, and considering lifestyle implications. Physician-scientists, including MD-PhD and other dual-degree graduates, as well as MD graduates with research-intensive training, often pursue specialties with established research pathways. We outline key decision-making factors, including mentorship, clinical exposure, research commitment, and financial sustainability. Additionally, we compare research track and categorical residency pathways, detailing differences in training structure, funding opportunities, and career outcomes. The article explores the evolving role of physician-scientists across career stages, from residency through senior faculty leadership, highlighting strategies to maintain research engagement while balancing clinical responsibilities. By critically evaluating these factors and leveraging mentorship and institutional support, physician-scientists can make informed decisions that align with their aspirations, ensuring a fulfilling and impactful career in both medicine and research.

## Introduction

Choosing a clinical specialty is one of the most significant decisions in the career of a physician-scientist. This choice determines the nature of your clinical practice and profoundly influences your research trajectory and overall professional fulfillment. The journey to this decision is complex and multifaceted, requiring a deep understanding of your interests, skills, and long-term goals. As a physician-scientist, you are uniquely positioned at the intersection of clinical care and scientific discovery (National Institutes of Health, 2014). This dual role offers the opportunity to make groundbreaking contributions to medicine, but it also demands a careful balance between clinical duties and research activities (Wyngaarden, 1979; Ley and Rosenberg, 2005). Importantly, the ability to sustain a physician-scientist career is often determined as much by the institutional environment and training program as by the specialty itself (Bradford et al., 1996).

Selecting a specialty is profoundly personal and requires introspection and reflection. It involves considering your passions and interests, evaluating your strengths and skills, and understanding the lifestyle implications of different specialties. Additionally, seeking mentorship and gaining hands-on clinical exposure are crucial steps in making an informed decision. Your chosen specialty will shape your career path, influence your daily work, define your colleagues, and impact your ability to contribute to medical science (Zemlo et al., 2000; Hauser and McArthur, 2006).

**Figure 1.**
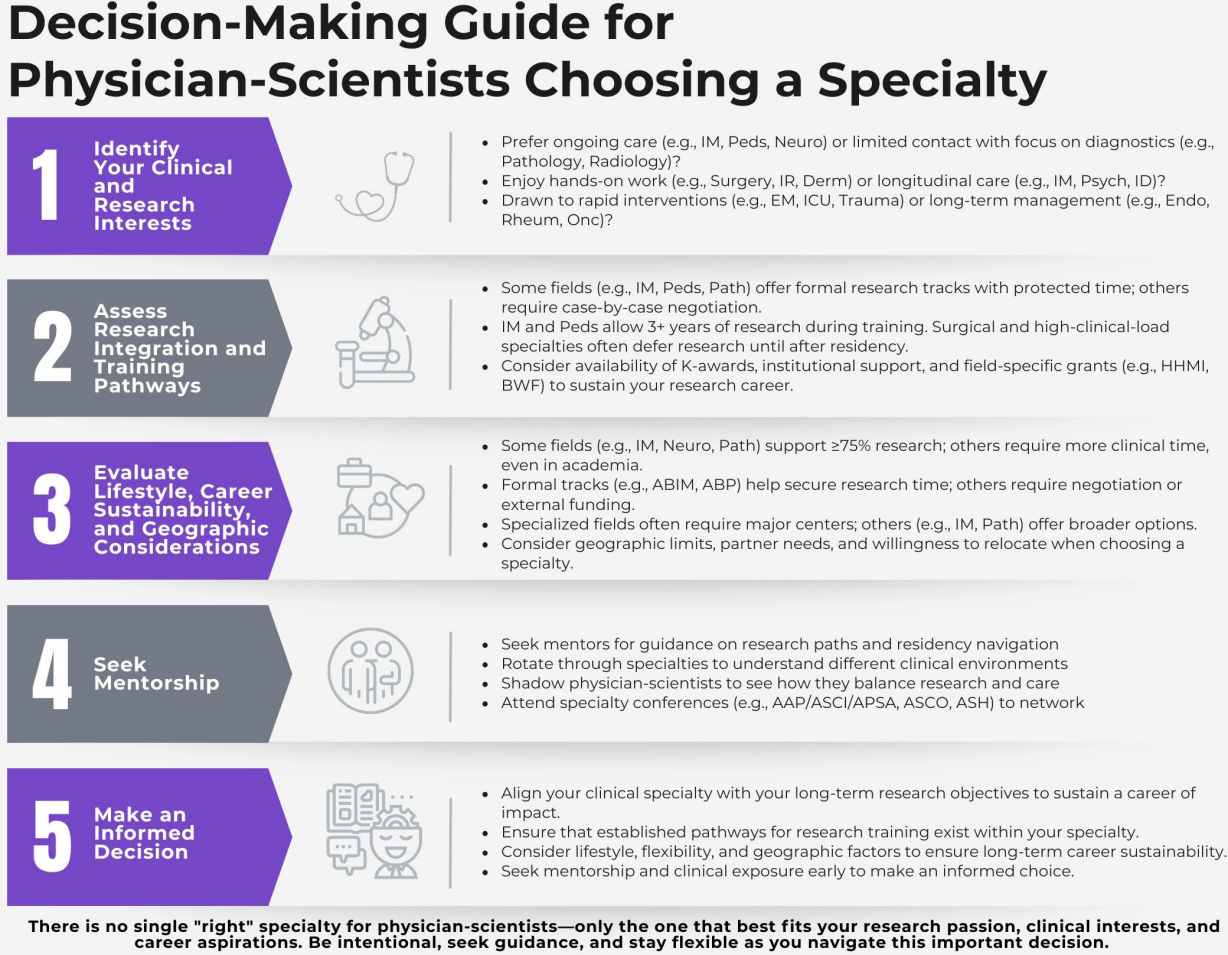
Decision-making framework for physician-scientists choosing a clinical specialty. This figure presents a structured approach to specialty selection for physician-scientists, highlighting key considerations that inform an intentional and sustainable career choice. The framework emphasizes alignment of clinical and research interests, evaluation of research integration and training pathways, assessment of lifestyle and career sustainability factors, engagement with mentorship, and synthesis of these elements to support informed decision-making.

Across these considerations, five factors repeatedly shape long-term physician-scientist success: alignment between clinical and research interests, the structure of clinical work, availability of mentorship and research pathways, institutional culture, and financial sustainability. Figure 1 provides a structured decision-making framework to help physician-scientists evaluate clinical specialties. To complement this framework, Table 1 distills the key factors that physician-scientists should prioritize when comparing specialties and training environments. The following sections expand on these considerations and provide a structured framework for specialty selection. Because everyone’s experiences and goals are unique, trainees should seek guidance from physician-scientist role models across specialties as well as from training pathway directors, residency directors, and MD-PhD program leaders.

**Table 1.** Key decision factors for physician-scientists choosing a clinical specialty.

Factor	Why it matters	What to look for	Potential red flags
Alignment of clinical specialty with research focus	Sustained success as a physician-scientist depends on the ability to generate clinically informed research questions and maintain access to relevant patient populations, tissues, or data	Clear conceptual or translational link between clinical field and research area; access to relevant patient cohorts, clinical trials, or biospecimens; established examples of physician-scientists in that field	Difficulty accessing relevant patient populations or data; research that is only tangentially related to clinical work; reliance on external collaborators for core aspects of research
Structure of clinical work and Its impact on research time	The intensity, unpredictability, and procedural demands of clinical practice directly influence the ability to sustain protected research time	Predictable or modular clinical schedules; ability to consolidate clinical time; flexibility in clinical effort; specialties with established norms supporting reduced clinical load for research-focused faculty	High procedural volume requirements to maintain competency; unpredictable call schedules; continuous longitudinal patient responsibilities that fragment research time; inability to reduce clinical effort
Availability of structured research pathways and mentorship	Early-career success is strongly influenced by access to structured training pathways and experienced physician-scientist mentors	Formal research tracks (e.g. ABIM Research Pathway, Holman Pathway); multiple NIH-funded investigators in the department; strong track record of trainees obtaining K awards and transitioning to faculty positions; culture of mentorship	Reliance on a single mentor; absence of structured research pathways; limited history of trainee success in research careers; lack of grant-writing or career development support
Institutional environment and culture	Institutional commitment is often more determinative than specialty alone in enabling long-term success as a physician-scientist	Departments with multiple R01-funded investigators; presence of T32 training grants; institutional bridge funding and startup support; leadership that values and protects physician-scientist careers; collaborative research environment	Departments primarily driven by clinical revenue (RVUs) with limited research infrastructure; lack of protected time enforcement; minimal NIH funding; absence of physician-scientists in leadership roles
Financial model and long-term sustainability	Financial pressures influence career decisions, effort allocation, and retention in research	Transparent compensation models that support protected research time; startup packages, bridge funding, and salary guarantees; access to loan repayment programs (e.g. NIH LRP); alignment between clinical effort and research expectations	Heavy reliance on clinical revenue to support salary; misalignment between expected research effort and financial support; lack of institutional investment in early-career investigators; financial pressures that incentivize abandoning research

## Understanding the landscape: trends and training pathways

### Trends in physician-scientist training specialty choice

Physician-scientist trainees often gravitate toward certain medical specialties that align well with research activities (Table 2). According to a 2008 study published in JAMA, the most popular specialties among MD-PhD graduates were Internal Medicine, Pediatrics, Surgery, Pathology, Neurology, and Radiology (Andriole et al., 2008). A 2023 study evaluated 11 specialties over 11 years (2009–2019) and found that most US allopathic MD-PhD graduates entered internal medicine, followed by pediatrics and pathology (Emala et al., 2023). When normalized to the relative size of total residency positions, the smaller residencies, such as neurosurgery (7.2%), pathology (6.4%), and neurology (3.3%), had the highest percentage of entering residents who were MD-PhD graduates (Emala et al., 2023). More recent data suggest that some additional residency specialties are gaining popularity among MD-PhD graduates, including Dermatology, Anesthesiology, and Psychiatry. These fields typically provide structured opportunities to integrate research with clinical practice. Even nontraditional research specialties, such as Family Medicine, Emergency Medicine, Hospital Medicine, and Sports Medicine, are increasingly developing institutional pathways to support research careers. However, these pathways are less common among MD-PhD graduates (AAMC, 2023).

**Table 2.** Physician scientist specialty choices, research integration, and funding opportunities across residency and fellowship training.

Specialty	% of MD-PhD grads (Akabas and Brass, 2019)	Structured research program during residency/fellowship	Nature of clinical practice	Research potential post-training	Common research areas
Internal medicine	27%	ABIM Research Pathway (https://www.abim.org/certification/policies/research-pathway/policies-requirements/)	Broad, inpatient/outpatient, chronic disease management	Strong NIH funding (K/R awards), academic research careers well-established, ABIM physician-scientist pathway	Immunology, infectious diseases, cardiovascular disease, cancer biology, respiratory diseases, GI pathophysiology
Pediatrics	13%	ABP Integrated Research pathway (https://www.abp.org/content/integrated-research-pathway-irp)	Outpatient and inpatient, developmental and longitudinal focus	NIH-supported research pathways, subspecialty fellowships integrate research	Developmental biology, cancer biology, infectious disease, immunology, inborn errors of metabolism, genetics of inherited and rare diseases
Pathology	10%	ABPath Physician-Scientist Research Pathway (https://abpath.org/physician-scientist-research-pathway/)	Laboratory-based, diagnostics, molecular testing, typically both anatomic Pathology (AP, tissue diagnosis) and clinical Pathology (CP, clinical laboratory testing) but sometimes just one or the other is completed. CP can be the shortest of all residencies (two-years)	Lab-based research seamlessly integrates into clinical work; NIH and industry funding prevalent	Cancer biology, molecular diagnostics, immunopathology, broad pathophysiologic investigations
Neurology	9%	Many institutions offer research tracks which provide a combined residency program in Neurology and Psychiatry, emphasizing research training	Inpatient and outpatient, complex chronic disease focus, Neuro-ICU and procedural tracks exist, clinic-based	NIH funding for neuroscience, translational and clinical research, research-heavy fellowships available	Neuroscience, stroke, neurodegeneration, neuroimmunology, epilepsy, movement disorders
Radiology	5%	ABR Holman Research Pathway (https://www.theabr.org/diagnostic-radiology/initial-certification/alternate-pathways/holman-research-pathway). Some programs offer dedicated research tracks, often supported by NIBIB T32s	Technology-driven, imaging-based diagnosis, procedural approaches (interventional radiology (IR)) and targeted therapy (IR and nuclear medicine)	Research in imaging, imaging technology, AI applications, application of imaging to translational research, NIH, foundation, and industry funding sources available	Imaging and image-guided therapy methods, biomedical engineering, applied physics and chemistry, molecular imaging and therapy, applications of imaging to specific disease biology
Psychiatry	5%	Many institutions offer specialized training within general residency dedicated to nurturing future clinical and basic neuropsychiatric researchers.	Outpatient-focused, long-term patient care	NIH (NIMH) funding opportunities for neuropsychiatric research; research-focused career pathways	Neuroscience, neuropsychiatric disorders, addiction research
Surgery (all)	12%	Some programs offer dedicated research years or tracks, though not universally standardized.	Procedural, high-intensity, acute care	Limited protected research time; research opportunities available but require leadership negotiations for protected time and effort to integrate into career. For NIH K awards, surgeon-scientists with active surgical duties can request a reduced effort of 50%	Transplantation/Immunology, surgical oncology, regenerative medicine, trauma/acute care/sepsis, neurosurgery, vascular surgery, burn/wound healing
Medical genetics	1%	ABMGG (https://www.abmgg.org/initial-certification/certification-pathways/)	Consultation-based, highly specialized. Often combined with medicine, pediatrics, or Ob/Gyn, sometimes neurology and pathology	Strong NIH and industry support; translational genomics and precision medicine research expanding rapidly	Genomics, rare disease research, precision medicine
**Derm.**	3%	Certain institutions offer research-focused fellowships or integrated research tracks during residency.	Outpatient with inpatient consult opportunities. Opportunity for small and large procedures. Average practice has a mix of cutaneous oncology, immunology, autoimmunity, and aesthetics	NIH, foundation, and industry funding available, including CDAs; translational research opportunities exist	Immunology, microbiology, skin cancer, neurobiology, stem cell and stromal cell biology, AI, epidemiology, health disparities
Radiation oncology	4%	Many programs offer dedicated research time or tracks; specifics vary by institution (https://www.acgme.org/specialties/the-holman-pathway).	Procedural, oncology-focused, technology-driven	NIH and private funding for cancer research; translational/clinical research integrated into practice.	Cancer biology, radiation therapy research
Family medicine	<1%	Limited Formal Research Tracks: Research opportunities may be available but are often less structured compared to other specialties.	Outpatient primary care, broad-spectrum medicine	Few research-intensive career pathways; NIH funding is uncommon.	Community health, health disparities, preventative medicine
Ob/Gyn	<1%	Some institutions offer research tracks or fellowships focusing on areas like maternal-fetal medicine.	Procedural and medical, maternal-fetal focus	Research exists in maternal-fetal medicine and gynecologic oncology, but protected research time is limited.	Reproductive biology, maternal-fetal medicine, gynecologic oncology
Ophthalm.	4%	Some programs offer integrated research training during residency; availability varies by institution.	Procedural, vision-focused, outpatient	NEI (NIH) funding strong; vision science research well-supported.	Vision science, retinal disease, corneal research

### Residency and Fellowship research tracks: structured and institution-specific pathways

Physician-scientists pursuing research-intensive careers benefit from structured research tracks embedded within residency and fellowship programs (Table 3). While some specialties have well-established national programs (e.g. ABIM Research Pathway, ABP Accelerated or Integrated Research Pathway, ABPath Physician-Scientist Research Pathway in Pathology; the Holman Pathway in Radiology and Radiation Oncology), others rely on institution-specific tracks that are not always widely advertised, vary in research time and resources, and are structured heterogeneously, requiring individual investigations into programs of interest.

**Table 3.** Comparison of research track and categorical residency pathways using internal medicine as an example.

Key features	Research track	Categorical track
Primary focus	Balances clinical training with early transition to protected research time	Primarily focused on clinical training
Structure	Integrates significant research time within fellowship; protected time for research built into the training plan. Includes built-in mentorship with clear expectations for faculty transition. Research-focused institutions often provide structured pathways leading to junior faculty positions	Full-time clinical training with optional research electives
Duration	2 years of residency, 3+years of fellowship (with embedded post-doctoral training)	3 years of residency, 3 years of fellowship
Eligibility	Requires strong research experience and a clear commitment to a physician-scientist career. Board eligibility for Internal Medicine in PGY-4 or PGY-5, subspecialty boards in PGY-6 or later	Open to all applicants, requires completion of standard residency and fellowship clinical requirements before board certification
Common specialties	Internal Medicine (ABIM Research Pathway), Pediatrics, Neurology, Pathology, Radiology	All medical specialties
Research Commitment	≥80% required research effort during later fellowship to early faculty years	Usual ~80% research effort for ~1 year
Funding support	≥80% research effort during later fellowship years; often includes NIH R38, R25, F32, or T32 support with expectation of application for mentor career award at the end of the training	Centers for Medicare and Medicaid Services-based funding; research funding may be available for those pursuing academic careers
Career outcomes	High proportion of graduates enter academic medicine (physician-scientist careers, faculty roles, NIH-funded research)	Majority enter clinical practice, though some continue in academic medicine
Application considerations	Requires strong research experience, commitment to physician-scientist training, and alignment with institution’s research mission	Field-specific competitiveness features
Mentorship and support	Structured mentorship with dedicated physician-scientist training programs, institutional funding, and NIH support	Highly dependent on individual institution and faculty mentors
Effort and activities by PGY year (sample based on Internal Medicine timeline)		
PGY1	Intern year – 100% clinical responsibilities, inpatient-heavy	Intern year – 100% clinical responsibilities, inpatient-heavy
PGY2	Resident (first year) ~80–90% clinical, 10–20% research (depending on program structure); initial research planning Fellowship match	Resident (first year) ~80–90% clinical, 10–20% research (depending on program structure) with most having some scholarly requirement
PGY3	Fellowship year 1 100% clinical subspecialty training board-eligible for IM boards	Resident (second year) ~80–90% clinical, 10–20% research (depending on program structure), most with scholarly requirement fellowship match board-eligible for IM boards
PGY4	Fellowship year 2 80% research <20% clinical	Fellowship year 1 100% clinical subspecialty training
PGY5	Fellowship year 3 80% research <20% clinical board-eligible for subspecialty boards	Fellowship year 2 80% research <20% clinical board-eligible for subspecialty boards
PGY6	Fellowship year 4 80% research <20% clinical	Fellowship year 3 (80% research <20% clinical)
PGY7	First faculty or other position – requires 80% research commitment for NIH career development awards (e.g. K08, K23, K99/R00)	First faculty or other position

#### Institution-specific research pathways

Certain specialties—including Neurology, Psychiatry, Dermatology, Emergency Medicine, Radiology, Radiation Oncology, Surgery, and Anesthesiology—have evolving or institution-specific physician-scientist pathways that provide dedicated research time. These programs vary by institution but often include:

- Flexible training structures that integrate 12–24 months of protected research during or after clinical training.
- Specialized mentorship programs linking residents with established physician-scientists.
- Early access to K-award preparation and grant-writing support. (including institutional awards through local NIH-funded Clinical and Translational Science awards).
- Additional funding mechanisms from departmental T32 training grants, foundation fellowships, or institutional pilot grants.

#### Joining a research track after starting residency

Not all physician-scientists enter residency on a designated research track. Many programs allow residents to transition into research tracks after starting clinical training, creating opportunities for trainees whose interest in a research career develops later in residency. When considering switching into a research track, one should:

- Network within your department early in residency to express interest in research opportunities.
- Seek mentorship from physician-scientists who can advocate for your transition into a research-intensive pathway.
- Apply for internal institutional funding to support research during residency or fellowship.
- Utilize NIH R25, R38, F32, K99/R00, or foundation fellowships to carve out research time during clinical training.

If you are applying to residency and considering research, inquire with programs about their flexibility in accommodating physician-scientists outside formal research pathways before ranking them. Some programs/residencies strongly support research flexibility, while others require early commitment to a structured track.

## Self-assessment: aligning clinical specialty with research goals

Building on the key decision factors outlined in Table 1, the process of selecting a clinical specialty for physician-scientists can be approached as a structured self-assessment of interests, strengths, priorities, and long-term goals.

### Reflect on your priorities, interests, and passions

The journey to selecting a clinical specialty begins with introspection. For physician-scientists, this process should be anchored in a clear understanding of the type of research you want to pursue and the amount of time you intend to devote to it. Because your clinical specialty will shape and ideally strengthen your research program, alignment between clinical work and scientific inquiry should be a primary consideration when choosing a specialty.

From this perspective, reflect on the clinical experiences that have most excited you and that best support your long-term research goals. Consider what you find most compelling about medicine: the pathophysiology of disease, the laboratory and diagnostic aspects of care, the medical or surgical treatment of illness, or some combination. Do you enjoy procedures, which may align with surgical, anesthesiology, or interventional subspecialties? Are you drawn to inpatient, outpatient, critical care, or operating room environments? Consider the types of patients, diseases, and clinical problems that sustain your curiosity and engagement.

A specialty that complements your research can provide a cohesive and fulfilling career path, allowing you to integrate clinical insight with scientific discovery (Glowinski et al., 1998; Kosik et al., 2014). For example, a focus on cancer biology may align naturally with oncology, while an interest in neural systems may align with neurology, psychiatry, or neurosurgery. Studies suggest that MD-PhD graduates who select specialties aligned with their research are more likely to sustain research engagement over time (Milewicz et al., 2015). At the same time, less traditional research specialties—including surgery, anesthesiology, and emergency medicine—can also support meaningful integration with focused research questions.

As you synthesize your interests, strengths, and long-term goals, it is important to choose a specialty (and, where relevant, a subspecialty) that not only aligns with but actively strengthens your research program. Importantly, while alignment is central to specialty selection, the structural feasibility of sustaining research varies across fields and is addressed in later sections.

It is also important to take a forward-looking perspective. Trainees are often drawn to specialties at the forefront of technological innovation—for example, artificial intelligence in radiology, dermatology, or pathology. However, the landscape of any field may evolve significantly by the time you complete training and establish independence. Selecting a specialty in which the patient population, underlying biology, and clinical questions will continue to inspire your work as your career evolves may be more important than focusing on any single emerging technology.

Finally, be mindful of the distinction between your student experience and the reality of long-term practice. Many trainees are energized by rotating with a highly engaged clinical team, but it is more important to reflect on your own reactions to the patients, diseases, and clinical problems encountered. You can build a strong and supportive clinical environment over time, but the patient populations and clinical focus of a specialty will remain central to your career.

### Evaluate your skills and strengths

In addition to considering your clinical interests and research goals, it is important to consider your individual strengths and aptitudes, which may influence both clinical fit and long-term success. In various clinical settings, you may excel in procedural skills, patient/family interactions, team leadership, and diagnostic challenges. Understanding your clinical strengths can guide you toward a specialty where you can thrive. For example, you might consider a surgical specialty if you enjoy performing intricate procedures. Those with high visual aptitude or who enjoy pattern recognition might gravitate toward Dermatology, Pathology, or Radiology.

On the other hand, if you excel in patient relationships and long-term management, a specialty such as Internal Medicine, Neurology, Psychiatry, or Pediatrics might be more suitable (Patel et al., 2012). If understanding disease pathophysiology is your passion, Pathology might be a good choice. Similarly, consider how your research skills can be integrated into your clinical practice. Some specialties may offer more opportunities for translational research, where you can apply findings from basic science to clinical settings, and vice-versa. For example, the frequency and ease of procedures that yield healthy or diseased clinical tissue specimens vary by specialty and subspecialty; adult skin biopsies are easier to obtain than pediatric liver biopsies. The success of physician-scientists often hinges on their ability to integrate clinical expertise with research, as studies show that early-career engagement in research-intensive specialties correlates with sustained NIH funding and academic careers (Marsh and Todd, 2015). Keep in mind that several sub-specialties within Internal Medicine, Pediatrics, Surgery, Anesthesiology, and Neurology might allow you to practice in a procedural or intensive care subspecialty. At the same time, it may also allow you to maintain an exclusively clinic-based practice.

### Consider lifestyle, geography, and work-life integration

Beyond interests and skills, the structure of clinical work and its impact on daily life are critical considerations in selecting a specialty. Different specialties come with varying demands on your time. Considering how the typical work hours and on-call responsibilities align with your personal life and well-being is essential. Trainees should be realistic about the possibility that periods of intense clinical responsibility may temporarily deprioritize research activities. For instance, some specialties, such as Surgery and surgical and procedural subspecialties, may require more clinical hours to maintain procedural proficiency, including frequent on-call duties and urgent procedures. Conversely, specialties such as Dermatology, Pathology, Emergency Medicine, and Anesthesiology, or Radiology often offer more predictable schedules. Work-life balance, including caregiving and personal responsibilities, is influenced not only by clinical schedules and demands but also by departmental culture and the degree of collegial and institutional support for flexibility and protected leave. These factors should be considered when balancing clinical, research, and personal commitments (Culican et al., 2014). Depending on the nature of your research interests and preferred work style, uninterrupted time away from call responsibilities may be especially valuable.

Think about where you want to live and work. Some specialties may offer greater flexibility in practice settings and locations, allowing you to better balance your professional and personal life. For example, if you prefer to work in an urban academic center, specialties with strong academic and research components, such as Oncology or Cardiology, might be more appealing. On the other hand, if you envision practicing in a rural or community setting, general Internal Medicine, Pediatrics, or other primary care specialties might be a better fit; however, research opportunities may be very scarce, so this option should be approached with great care.

### Length of training and timing of research independence

In parallel with lifestyle considerations, the length and structure of training are important factors shaping the timing of research independence and career progression. Indeed, training duration is a critical factor to consider, especially for physician-scientist graduates who have already invested significant time in their education. The duration of residency and fellowship training varies widely across specialties, and this can have long-term implications for both career trajectory and personal well-being, especially for those who have already invested in combined MD/PhD training. Residency in fields such as Internal Medicine, Pediatrics, or Pathology may provide a faster route to research independence than longer training pathways in surgical or other highly procedural specialties, although for many physician-scientists, alignment between specialty and research program ultimately outweighs training length alone.

For those eager to establish a research career early, choosing a specialty with a shorter training period and structured research pathways may enable a smoother transition to a faculty position and access to grant funding. Conversely, if a more extended training period aligns with your passion and career goals, it may be worthwhile despite the added commitment. Ultimately, understanding the balance between training length, clinical responsibilities, and research opportunities can help you make an informed decision that supports both professional fulfillment and personal sustainability. It is essential to recognize that excellent clinical training is a foundation for a career as a physician-scientist. The commitment to research during the early faculty years means that the continued growth in clinical judgement and experience of a physician-scientist will not be the same as that of a full-time clinician in the same specialty. Thus, it is critical not to shortchange clinical training during residency, which will be the foundation of excellence in patient care throughout one’s career. Keep in mind that the journey in medicine is as important as the destination, and rewarding experiences will inform your research and help you refine your thinking—even when those experiences are diverse and multidisciplinary.

### Seek mentorship and advice

While self-assessment is essential, informed decision-making also depends on guidance from experienced mentors and exposure to real-world clinical environments. Mentorship is invaluable when choosing a specialty. Seek out physician-scientist mentors across fields who can offer practical insight and career guidance. Many clinicians are willing to discuss their career paths and may also be open to arranging shadowing opportunities. Mentors can help you understand the realities of different fields and offer valuable advice. Networking is also crucial. Attend conferences, workshops, and specialty interest groups to connect with professionals in your areas of interest. These interactions can provide a deeper understanding of different specialties, the values of colleagues you will eventually join, and help you make a more informed decision (Brass et al., 2010). Additionally, mentors can introduce you to key figures in the field, provide opportunities for collaboration, and offer support as you navigate your career path (Schafer, 2009).

### Gain clinical exposure

In addition to mentorship, direct clinical exposure across specialties provides critical insight into the day-to-day realities of practice. Hands-on experience is essential in making an informed decision about your specialty. Take full advantage of clinical rotations and electives to gain exposure to different specialties. This experience can help you understand the day-to-day activities and patient interactions in various fields. For example, a rotation in Pediatrics can provide insight into the unique challenges and rewards of working with children. At the same time, an elective in Neurology can provide insights into the complexities of diagnosing and treating neurological disorders. An elective in Pathology would reveal that day-to-day practice differs significantly from your experience in the Pathology course, involving the diagnosis of numerous diseases and regular interaction with a diverse range of clinicians and researchers. Additionally, spend time working with physician-scientists in different specialties. Observing their work can provide valuable insights into the realities of each specialty and help you determine which one aligns best with your interests and goals. Shadowing can also help you understand the workflow, patient demographics, and common procedures for each specialty, providing a more comprehensive view of what to expect. Most of all, remember that being a specialist in a field is different from being a student on that clinical service—try to understand what mentors’ practice looks like and how it interfaces with your scientific career.

### Reflect on long-term goals and career satisfaction

As these factors are considered together, it is equally important to take a broader, long-term perspective on career development and satisfaction. Consider how your chosen specialty will sustain your intellectual engagement and professional fulfillment over the long term. A physician-scientist career unfolds over decades, and the clinical and scientific questions that motivate you should remain compelling as your interests evolve. Some specialties are undergoing rapid transformation with the introduction of new technologies and therapies, creating opportunities for innovation, while others offer deep opportunities for collaboration and sustained inquiry. Reflect on the types of problems you want to solve and the impact you hope to have on medicine and research over time.

At the same time, it is important to recognize that a specialty choice is not irreversible. Many physician-scientists refine their focus during or after residency, either by pursuing different subspecialties or by evolving their research direction. Understanding that career trajectories are flexible can reduce the pressure to make a ‘perfect’ decision early and allows trainees to focus instead on building a strong and adaptable foundation.

An important consideration is that PhD training primarily teaches you how to think and conduct research, rather than limiting you to the specific topic of your thesis. The conceptual frameworks, methodological approaches, and problem-solving skills you develop can be applied across a wide range of clinical and scientific domains. For example, training in molecular pathways in one disease area may readily translate to investigations in another. As scientific fields and technologies continue to evolve, the ability to adapt and acquire new skills becomes far more important than the specific subject of one’s early work.

By focusing on a specialty that sustains your curiosity and supports continued intellectual growth, you can build a career that remains both meaningful and adaptable over time. Once you have identified a general clinical and research direction, the next step is to consider how training structures, institutional culture, and available resources will enable—or limit—your ability to sustain a dual career.

## Sustaining a dual career: research, clinical work, and institutional support

While the preceding sections focus on how trainees select a specialty based on interests and goals, sustaining a physician-scientist career depends on structural factors that extend beyond individual preference.

### Balancing clinical and research responsibilities

A physician-scientist career requires a careful balance between clinical duties and research activities. The ability to balance clinical and research responsibilities is strongly shaped by the structural demands of different specialties. Specialties such as Internal Medicine, Neurology, Pediatrics, and Pathology have historically provided more structured research pathways. Some specialties, such as anesthesiology and emergency medicine, have more clearly defined clinical schedules, which may facilitate balancing research commitments and personal responsibilities, compared with fields that require continuous longitudinal patient care and ongoing availability. In contrast, surgical fields often pose challenges in maintaining protected time for research, in part due to the need to maintain a volume of clinical cases to maintain procedural skills and the need for urgent procedures with less predictable operating room schedules. In practice, specialties with high procedural demands and unpredictable clinical schedules are often more challenging environments for sustaining research-intensive careers unless strong institutional protections are in place. While successful physician-scientists exist across all specialties, the structural ease of sustaining a research-intensive career varies substantially by field, and trainees should approach certain specialties with a clear understanding of the additional negotiation and institutional support required.

It is important to understand that your primary role as a physician-scientist is to advance medical knowledge through research. This means that a substantial amount of your time will be spent designing studies, conducting experiments, analyzing data, publishing findings, grant-writing and rewriting, mentoring, teaching, and training. *You should strive for a clinical practice that informs and enhances your research, and vice versa*. The real-world challenges and patient interactions you encounter can inspire research questions and drive your scientific inquiries (Ley and Rosenberg, 2005). For example, observing a particular complication in patients with a specific disease might lead you to investigate its underlying mechanisms in the lab. This synergy between clinical practice and research can significantly advance both fields. It is important to recognize that achieving this balance may evolve over time, particularly during the mentored phase of a physician-scientist’s career, when research independence, protected time, and clinical roles are still being established.

### Protected time, institutional culture, and specialty norms

Protected time is critical for physician-scientists, enabling meaningful scientific investigation while maintaining clinical responsibilities. Some specialties, such as Internal Medicine, Neurology, and Pediatrics, have well-established pathways that integrate research into training, often through structured programs like the American Board of Internal Medicine (ABIM) Research Pathway or equivalent research tracks in other specialties. Additionally, these specialties are well supported by NIH funding mechanisms, such as K-awards and T32 training grants, which incentivize institutions to provide protected time for physician-scientists (Marsh and Todd, 2015). However, not all specialties offer the same level of institutional support for integrating research with clinical practice. While some training programs, particularly in fields such as Surgery, include built-in research years and T32-supported research positions, the level of support can vary significantly across institutions. It is essential to recognize that a training program’s research orientation may not always align with the realities of practice in that field. Therefore, when choosing a specialty, physician-scientist trainees should consider both the institution-specific training opportunities and their long-term career goals regarding clinical practice and research. Institutional support, NIH K-awards, and departmental policies play a critical role in ensuring that physician-scientists remain engaged in research. Data suggest that protected time early in a physician-scientist’s career correlates with long-term success in NIH funding, underscoring the importance of evaluating a specialty’s culture and infrastructure for physician-scientist development (Hauser and McArthur, 2006). When choosing a specialty, physician-scientist graduates should consider how research time is structured during residency and fellowship and whether their chosen field has mechanisms to shield research commitments from clinical encroachment. Furthermore, different training programs within a field offer varying research opportunities and support, and trainees should inquire explicitly about these differences during the residency and fellowship application process.

When evaluating residency and fellowship programs, trainees should also assess the broader institutional environment for physician-scientists—not just research time during training, but also faculty-level commitments to career development and grant stability. One metric that may be useful for assessing an institution’s commitment to research is the amount of NIH funding it secures, both for research and training grants. Trainees can further evaluate departmental strength by examining: (i) the number of R01-funded investigators within the department, (ii) the presence of institutional training grants (e.g. T32 programs), and (iii) the track record of trainees transitioning from mentored (K) awards to independent (R) funding. Resources such as the Blue Ridge Institute for Medical Research and NIH RePORTER can facilitate this assessment.

The availability of protected time for research varies widely across specialties, driven by differences in clinical workload, funding structures, institutional priorities, and historical precedent. However, this is highly dependent on institutional culture. While some procedural and highly clinical fields, such as Surgery, Anesthesiology, and Emergency Medicine, might traditionally be perceived as having fewer built-in mechanisms for protected research time, many have developed strong pathways and a track record of success. In fact, the structured nature of shift work in some of these specialties can be advantageous for those intending to balance clinical duties with research. When off duty, physicians in these fields often have uninterrupted time to dedicate to research.

Revenue models that favor clinical procedures can disincentivize research (because clinically supported time generates much more revenue in these specialties than research grant funding). Conversely, specialties that incorporate generously reimbursed but short procedures can, in the right financial model, enable physician-scientists to subsidize research effort even with less clinical effort. Ultimately, the ability to integrate research with clinical practice in these specialties often hinges on the institution’s specific culture and infrastructure (Garrison and Deschamps, 2014). As a result, physician-scientists in these fields usually must negotiate protected research time individually, secure external funding, or pursue alternative pathways, such as research sabbaticals or post-residency research fellowships.

Moreover, the culture within a specialty can strongly influence how research is valued. Fields and departments with a strong tradition of clinician-investigators tend to embed research expectations into career development. In contrast, specialties that prioritize technical proficiency and procedural expertise may view research as secondary to clinical mastery. While some physician-scientists have successfully established research careers in traditionally non-research-intensive specialties, doing so often requires additional advocacy, mentorship, and institutional support (Culican et al., 2014). Physician-scientist graduates should carefully evaluate these dynamics and seek informed mentors when selecting a specialty, considering not only their research interests but also the structural support available for sustaining a dual career. These considerations reflect several of the structural factors highlighted in Table 1, particularly the importance of institutional environment and clinical workload in shaping long-term research sustainability.

Taken together, these factors reinforce that institutional environment and departmental culture are often as determinative as specialty choice itself in shaping a sustainable physician-scientist career.

### Financial considerations and long-term sustainability

The financial realities of a physician-scientist career are essential, but often undiscussed, factors to consider when selecting a specialty. While dual-degree training offers unique opportunities for impactful research and leadership, it may entail trade-offs in earning potential and financial stability relative to full-time clinical peers. Physician-scientists typically enter the workforce later than their MD-only counterparts, often after 7–10 years of additional training beyond medical school. This delay affects lifetime earning potential, as does the expectation of devoting a substantial portion of time to research rather than full-time clinical practice (Catenaccio et al., 2024). A recent analysis comparing MD-PhD and single-degree MD academic physicians found that MD-PhD graduates earn a median of $363,655 less over their careers, representing a 7% reduction in lifetime earnings (Catenaccio et al., 2024). This gap is even more pronounced in procedure-heavy specialties such as Neurosurgery, where MD-PhD graduates earn more than $1.8 million less over their lifetimes than their MD counterparts. However, it is important to weigh this difference against the equally long-term difference in personal fulfillment and professional freedom associated with a career as a physician-scientist. MD-PhD physicians thus tend to select clinical specialties that allow more research time, rather than those that maximize financial revenue. However, the greater, and generally unrecognized, challenge lies in defining one’s financial goals over time. This is a complex decision that is often heavily shaped by one’s immediate financial needs and sense of perpetually deferred compensation. Although occasional entrepreneurial success may offset some of these financial tradeoffs, most physician-scientists will benefit more from developing a clear and informed set of long-term financial expectations. Doing so can: (i) serve as a powerful negotiating tool to help align, rather than pit, one’s scientific, clinical, and financial interests against one another and (ii) avoid more perilous comparative or limit-testing approaches.

Notwithstanding the preceding considerations of financial needs and goals, it is equally important to recognize that, unlike clinicians in high-revenue specialties, physician-scientists often earn less than their full-time clinical peers due to the reduced revenue associated with decreased patient care responsibilities and dependence on external grant funding as their primary source of revenue (Catenaccio et al., 2024). External financial pressures—such as family obligations and the high cost of living in some communities—can thus influence career decisions (Jansen et al., 2023). In fact, studies suggest that financial concerns contribute to attrition from research careers, particularly for those facing funding instability post-training (Swartz et al., 2024). In contrast, physician-scientists in high-paying fields are likely to devote less time to research than those in lower-paying fields, where the salary disparity is less pronounced (Catenaccio et al., 2024).

Because research funding is variable while salaries are relatively fixed, the choice of institution and the level of institutional support for physician-scientists are vital but underrecognized financial considerations. For physician-scientists, even grant-funded research time incurs costs borne by individual departments. Many academic centers offer startup packages or bridge funding to physician-scientists transitioning into faculty roles, but this support often continues even for those further along in their careers. These packages may include:

- Guaranteed protected research time (e.g. 75% research time for the first 3–5 years)
- Internal pilot grants to support preliminary data collection
- Salary guarantees to offset gaps in extramural funding
- Research Support, also known as Development Funds or ‘Start-up Package’. The amount will vary based on several factors. It is critical to seek input from established investigators in your field when negotiating this component, which may support research assistants, reagents, animals, core services, and related research needs.

MD-PhD graduates generally have lower educational debt than MD graduates due to program funding (Andriole et al., 2008), but salary variability across specialties affects long-term financial security. Loan repayment programs can help mitigate financial pressures:

- NIH Loan Repayment Program (LRP) offers up to $50,000 per year in debt repayment for physician-scientists conducting NIH-funded research.
- Institutional loan forgiveness programs—some academic centers provide loan repayment incentives to attract physician-scientists.
- Public Service Loan Forgiveness (PSLF) applies to academic medicine roles at qualifying institutions.

Understanding how funding stability, institutional support, and salary structure vary across specialties is essential for making informed career decisions. Financial sustainability should be weighed alongside research passion and clinical interests to ensure long-term career fulfillment. While physician-scientists face financial trade-offs, strategic planning—including selecting research-friendly specialties, negotiating startup support, and leveraging loan-repayment programs—can help maintain economic stability throughout a successful career.

## Career flexibility and evolution

### Alternative research-focused career paths

While many physician-scientist trainees envision an academic career, a growing number transition into industry, government, or other sectors where research plays a central role; the leadership skills acquired during physician-scientist training also serve them well in these roles. As a case in point, the perennially ‘most admired’ chief executive officer (CEO) by Fortune 500 business leaders was physician-scientist Roy Vagelos, who served as department chair before leading pharmaceutical giant Merck for many years. These paths offer robust career options with different advantages:

- Pharmaceutical/Biotech Industry: roles in drug development, translational medicine, and regulatory affairs. Higher salaries likely require transitioning away from independent research.
- Government Research (NIH, etc.): not reliant on extramural grants, policy-driven research, opportunities for high-impact work in public health.
- Medical Technology and AI: growing opportunities in computational medicine, imaging informatics, and health-tech startups.
- Private Research Institutes and Foundations: positions at non-profits focused on disease-specific research (e.g. Broad Institute).
- Health Policy and Consulting: combining research expertise with strategy and policy work in firms like McKinsey or academic health systems.

For those interested in non-traditional careers, considering research-oriented specialties with broad applicability—such as Internal Medicine, Pathology, or Radiology—may provide greater flexibility.

### Career evolution over time

It is important to recognize that how you spend your time will change over the course of your career. In the beginning stages, you may spend more time building a sustainable research program, establishing your lab, and securing funding. This period is crucial for laying the foundation for your future research endeavors. As your career progresses, you might take on more administrative roles, become involved in education, or increase your clinical care responsibilities. For example, you might take on leadership roles within your department, mentor junior faculty and trainees, or develop new educational programs. This evolution is natural and can provide a diverse and enriching career experience. Mentoring motivated individuals is not only personally rewarding and intellectually stimulating, but also it can amplify the impact and breadth of your own research program. Planning for these changes, consulting with your mentors, and being adaptable can help you maintain a fulfilling and balanced career. Embracing these new roles can also create opportunities to shape your field and contribute to the broader medical community (Garrison and Deschamps, 2014).

Physician-scientists’ career trajectories evolve significantly from early training through mid-career and senior roles. Understanding these phases can inform specialty selection and long-term planning, as outlined in Table 4. Physician-scientists must actively manage their career evolution, ensuring they maintain research engagement while adapting to changing professional responsibilities.

**Table 4.** Career stages and key considerations for physician-scientists.

Career stage	Primary focus	Key challenges	Opportunities
Early career (residency, fellowship, first faculty position)	Establishing clinical competence, securing protected research time, and developing independent research projects	High clinical workload, obtaining first grants, managing dual training demands	Research-track residencies/fellowships (ABIM Research Pathway, Integrated Research Pathways in Pediatrics, Neurology, Pathology, Holman Pathway), NIH-funded career development awards (F32, K-awards)
Mid-career (first independent research grant to established investigator)	Balancing clinical, research, and administrative responsibilities; mentoring junior researchers; securing sustained funding	Time management, maintaining protected research time, transitioning to leadership roles	NIH R01 funding, leadership positions in research divisions, and industry collaborations
Late career (established investigator to senior leadership and mentorship)	Mentorship, institutional leadership, high-level advocacy for physician scientists	Sustaining research momentum, shifting toward administrative responsibilities, and succession planning	Directing training programs, serving on NIH study sections, and guiding institutional policy

## Conclusion

Remember that your extensive academic background embracing both clinical medicine and basic research truly sets you up to make significant contributions at the bench, at the bedside, and in between. Physician-scientists bring a unique and invaluable perspective to medicine, bridging the gap between scientific discovery and clinical application. Your ability to think critically, approach problems from multiple angles, and integrate research into patient care is rare and has a profoundly positive impact. Choosing a clinical specialty is a deeply personal decision that requires careful consideration of your interests, skills, lifestyle preferences, and long-term goals. This piece is intended to encourage reflection and discussion, recognizing that each individual’s experiences and goals are so unique that a comprehensive review cannot cover everything. It is essential to seek guidance from physician-scientist role models across various specialties, as well as from training pathway directors, residency directors, and MD-PhD program leaders. By reflecting on these factors, seeking guidance from mentors, and choosing both a specialty and an institutional environment that support your long-term goals, you can create a fulfilling and sustainable career that maximizes your impact in both clinical and scientific domains.

## Disclosures

THS is Senior Associate Dean for MD-PhD Education and Director of the Medical Scientist Training Program at the Icahn School of Medicine at Mount Sinai. Chair of the AAMC GREAT MD/PhD Section, Co-Chair of the AAMC GREAT Steering Committee, and a member of the Executive Committee of the National Association of MD/PhD Programs (now known as National Association of Clinician-Scientist Training). talia.swartz@mssm.edu.

JEC is Associate Dean for Research Training and has directed the South Texas Medical Scientist Training Program (currently T32GM145432) since 2011. He is past Chair of the AAMC GREAT MD/PhD section and past President of the National Association of MD/PhD Programs (now known as National Association of Clinician-Scientist Training). He directed the CTSA KL2 Junior Research Faculty Scholar program from 2014 to 2018, and has directed the ADRC Research Education Component (P30AG066546) at UT Health San Antonio since 2019. He served as program director of the ACGME-accredited Clinical Neurophysiology Fellowship for 18 years. He is also the Associate Dean for Research Training for Residents & Fellows since 2022. He is an elected fellow of the American Academy of Neurology, the American Neurological Association, the American Clinical Neurophysiology Society, and the American Epilepsy Society. cavazosj@uthscsa.edu.

MSH is Associate Dean for Physician-Scientist Education and has been the Director of the University of Washington Medical Scientist Training Program (T32GM153182) since 2008. He is a member of the Steering Committees of the AAMC GREAT Group MD/PhD Section and National MD/PhD Association, and an elected member of The American Society for Clinical Investigation (ASCI) and The Association of American Physicians (AAP). horwitz@uw.edu.

PJH is the Director, Medical Scientist Training Program (NIH T32GM149361) and Associate Dean for Medical Education at the University of Colorado School of Medicine. patrick.hu@cuanschutz.edu.

BS barbara.sampsonMD@outlook.com.

TCS is an Associate Director of the UCSF Dermatology Physician Scientist Training Program (Co-I: T325T32AR007175) and an elected member of The American Society for Clinical Investigation (ASCI). TCS is a Scientific Advisory Board member for Concerto Biosciences. tiffany.scharschmidt@ucsf.edu.

DAM is Associate Director for Education and Training in Penn’s Abramson Cancer (P30CA016520) and the Program Director for Penn’s Radiology Research Track PSTP (T32EB004311). david.mankoff@pennmedicine.upenn.edu.

AZ is the Associate Director, MD-PhD Training Program, University of Florida. He is a member of the AAMC GREAT Group MD/PhD Section Communications and Steering Committee. Ali.Zarrinpar@surgery.ufl.edu.

JC is the Director of the Physician-Scientist Residency Program and an Associate Director of the Medical Scientist Training Program at the Icahn School of Medicine at Mount Sinai. She is a multi-PI of the Mount Sinai StARR (Stimulating Access to Research in Residency) – NHLBI (R38HL172261) and Mount Sinai StARR – NIAID (R38AI181012). jaime.chu@mssm.edu.

CWE is the Director of the Virginia Apgar Society Research Track in Anesthesiology at Columbia University. He is the principal investigator for an NIH T32 training program in anesthesiology and serves as chair of the board of directors of the Foundation for Anesthesia Education and Research (FAER). cwe5@cumc.columbia.edu.

CSW is the Associate Dean for Physician Scientist Development, Director of the Medical Scientist Training Program (T32GM152284), co-PI of the Vanderbilt Stimulating Access to Research in Residency (StARR) (R38HL167237), Associate Director for Research Education, Vanderbilt Ingram Cancer Center, and former Director of the Internal Medicine Physician Scientist Training Program (PSTP) at Vanderbilt University. He is a member of the AAMC GREAT Group MD/PhD Section Steering Committee. He is on the Board of Directors of the American Physician Scientist Association (APSA) and the President of the National Association of MD/PhD Programs (now known as National Association of Clinician-Scientist Training [NACST]). Christopher.s.williams@vanderbilt.edu.
