# Supplementary material for: Navigating the path: Advice to physician-scientists on choosing a clinical specialty
Source: eLife. 2026 Jul 3;15:RP110448. doi: 10.7554/eLife.110448 (PMC13331548; doi:10.7554/eLife.110448)
Supplement: Peer review history

# Editor report: eLife Assessment

**DOI:** 10.7554/eLife.110448.3.sa0

**Authors:** Olujimi A Ajijola

**Affiliations:** University of California, Los Angeles United States

This Review Article provides a compendium of advice for MD-PhD students to consider when deciding which, if any, clinical field they will select for residency training. It is grounded in published data and effectively considers factors including the potential for clinical disciplines to sustain research integration, provide mentorship, meet lifestyle expectations, and foster a long-term career as a research-focused physician-scientist.

# Referee report: Reviewer #1 (Public review)

**DOI:** 10.7554/eLife.110448.3.sa1

[Editors' note: this version has been assessed by the Reviewing Editor without further input from the original reviewers. The review comments were minor and constructive, and the authors have been very responsive.]

Summary:

This brief piece by Swartz and colleagues outlines the complexities surrounding the choice of clinical specialty for physician-scientists. It is, in general, clear and well-written, and it will be useful to research-oriented medical students choosing a path and to the mentors who are guiding them.

Strengths:

The writing is clear. The points made are not profound, but they are important and will be of use to the intended audience.

# Referee report: Reviewer #2 (Public review)

**DOI:** 10.7554/eLife.110448.3.sa2

Summary:

This article is a useful compendium of advice for MD/PhD students (and research-focused MD students) to consider when it is time to decide on a clinical field for residency training. The authors are a distinguished group of physician-scientists and program directors who are drawing on published data and their own experience as mentors to provide advice and resources to students about to make what can be a career-defining choice. It makes an effective argument for considering important differences between clinical fields in their ability to sustain research integration, provide mentorship, meet lifestyle expectations, and foster a long-term career as a research-focused physician-scientist.

Strengths:

(1) A lot has been written about physician-scientists as an endangered species. Given the important role that physician-scientists can play if they engage in research that is informed by experience in patient care, not nearly enough has been written about the choices that students make during training that can keep them on track or throw them off.

(2) The article provides not only general advice, but specific information in the 2 tables that can help trainees to weigh their priorities and consider their options.

(3) Among the best advice is to weigh clinical demands, maintenance of procedural skills, recognition of the impact of research time on salary, and the impact of high salaries on the tension between research effort and clinical effort in clinical departments, which is where most physician-scientists in academia are employed.

# Author comment: Author response

**DOI:** 10.7554/eLife.110448.3.sa3

**Authors:** Talia Swartz, Jose E Cavazos, Marshall Horwitz, Patrick J Hu, Barbara Sampson, Tiffany Scharschmidt, Ali Zarrinpar, Nicholas Mohr, David Mankoff, Jaime Chu, Kyu Y Rhee, Charles W Emala, Christopher Williams

**Affiliations:** Icahn School of Medicine at Mount Sinai New York United States; UT Health San Antonio San Antonio United States; University of Washington School of Medicine Seattle United States; Vanderbilt University Medical Center Nashville United States; Office of the Medical Examiner of the City of New York New York City United States; University of California, San Francisco San Francisco United States; University of Florida Gainesville Gainesville United States; University of Iowa Des Moines United States; University of Pennsylvania Philadelphia United States; Icahn School of Medicine at Mount Sinai New York United States; Weill Cornell Medicine New York United States; Columbia University Irving Medical Center New York United States; Vanderbilt University Medical Center Nashville United States

The following is the authors’ response to the original reviews

**eLife Assessment**

This Review Article provides a compendium of advice for MD-PhD students to consider when deciding which, if any, clinical field they will select for residency training. It is grounded in published data and effectively considers factors including the potential for clinical disciplines to sustain research integration, provide mentorship, meet lifestyle expectations, and foster a long-term career as a research-focused physician-scientist.

We thank the editors for this positive assessment. We have revised the manuscript to sharpen the decision-making framework and make the advice more actionable, as detailed below.

**Public reviews:**

**Reviewer #1 (Public review):**

This brief piece by Swartz and colleagues outlines the complexities surrounding the choice of clinical specialty for physician-scientists. It is, in general, clear and well-written, and it will be useful to research-oriented medical students choosing a path and to the mentors who are guiding them.

We thank Reviewer #1 for these supportive comments.

Strengths:

The writing is clear. The points made are not profound, but they are important and will be of use to the intended audience.

We appreciate this assessment and agree that the value of this piece lies in consolidating practical, experience-based guidance in one resource for trainees and mentors.

Weaknesses:

I have only minor suggestions for improvement. There are some areas of redundancy where the article could be tightened up by consolidating.

We agree and have made substantial revisions to reduce redundancy throughout the manuscript. Specifically, we have streamlined the Introduction by removing a lengthy paragraph that previewed the article’s contents in a way that overlapped with later sections. The revised Introduction now concisely introduces five core decision-making factors (alignment between clinical and research interests, the structure of clinical work, availability of mentorship and research pathways, institutional culture, and financial sustainability) and directs readers to the new Table 1 and Figure 1 as organizing frameworks.

We have also consolidated overlapping discussions of research alignment, protected time, and clinical demands. The sections on clinical workload and protected research time have been tightened to minimize repeated points about specialty-specific demands, and we now cross-reference Table 1 rather than re-stating the same considerations in multiple places. Prose has been revised throughout for concision and clarity.

**Reviewer #2 (Public review):**

This article is a useful compendium of advice for MD/PhD students (and research-focused MD students) to consider when it is time to decide on a clinical field for residency training. The authors are a distinguished group of physician-scientists and program directors who are drawing on published data and their own experience as mentors to provide advice and resources to students about to make what can be a career-defining choice.

We thank Reviewer #2 for this generous and thoughtful evaluation.

Strengths:

(1) A lot has been written about physician-scientists as an endangered species. Given the important role that physician-scientists can play if they engage in research that is informed by experience in patient care, not nearly enough has been written about the choices that students make during training that can keep them on track or throw them off.

We share this perspective and appreciate the reviewer’s recognition of this gap in the literature. Our goal was precisely to address the decision-making process itself, which is often under-discussed in formal publications despite being a frequent topic in mentoring conversations.

(2) The article provides not only general advice, but specific information in the 2 tables that can help trainees to weigh their priorities and consider their options.

Thank you. We have further strengthened the tabular content in this revision by adding a new Table 1 (described below) and renumbering the original tables accordingly.

(3) Among the best advice is to weigh clinical demands, maintenance of procedural skills, recognition of the impact of research time on salary, and the impact of high salaries on the tension between research effort and clinical effort in clinical departments, which is where most physician-scientists in academia are employed.

We appreciate this feedback and have made this advice more prominent by incorporating these factors explicitly into the new Table 1 framework and by adding a more direct statement in the text about how specialty-specific structural differences affect the ease of sustaining a research career.

Area for Improvement

(1) Some of the most useful pieces of advice are scattered through the text when they might be more impactful if focused. For example, what are the 4 or 5 most essential factors that someone in an MD/PhD or an MD program should weigh when they are deciding between clinical disciplines? There are also published data on the experience of past graduates in achieving a research-focused career in each clinical discipline. How should that data be applied by trainees? What are the factors that should be weighed in deciding where to work as a research-focused physician once training has been completed?

We agree that the most critical decision-making factors were insufficiently distilled. To address this, we have made two major changes.

First, we have added a new Table 1: “Key Decision Factors for Physician-Scientists Choosing a Clinical Specialty.” This table identifies five essential factors—(i) Alignment of Clinical Specialty with Research Focus, (ii) Structure of Clinical Work and Its Impact on Research Time, (iii) Availability of Structured Research Pathways and Mentorship, (iv) Institutional Environment and Culture, and (v) Financial Model and Long-Term Sustainability—and for each provides columns describing Why It Matters, What to Look For, and Potential Red Flags. This table is designed to be directly actionable for trainees comparing specialties and programs.

Second, the Introduction now explicitly names these five factors as the organizing framework for the article and directs readers to Table 1 as a synthesis tool. The prior introductory paragraph, which previewed the article’s structure in a general way, has been replaced with a more focused synthesis.

Regarding the published outcomes data: we have retained the specialty-specific outcomes data in what is now Table 2 (previously Table 1) and have added context in the text about how trainees should interpret these data—specifically, that published graduation and career outcome data provide a useful baseline but should be weighed alongside institutional context, since the same specialty can look very different at different institutions.

Regarding factors for evaluating post-training positions: we have added a new paragraph in the section on Protected Research Time that addresses how trainees can evaluate the institutional environment at the faculty level, including specific metrics trainees can examine (see response to Points #4 and #5 below).

(2) Some clinical fields at academic institutions have proved to be much more hospitable to careers as research-focused physicians than others. Published data highlight the challenges. I believe the authors have tried very hard to present a balanced perspective, but in the process, they have, I believe, missed an opportunity to guide trainees and make them aware of what they should look for to avoid making a decision that may prove incompatible with their long-term goals.

We appreciate this candid observation and agree that our prior draft was overly cautious in this regard. In the revision, we have added a more explicit statement acknowledging that while successful physician-scientists exist across all specialties, the structural ease of sustaining a research-intensive career varies substantially by field. Specifically, we have added the following language to the section on Balancing Clinical and Research Responsibilities:

“In practice, specialties with high procedural demands and unpredictable clinical schedules are often more challenging environments for sustaining research-intensive careers unless strong institutional protections are in place. While successful physician-scientists exist across all specialties, the structural ease of sustaining a research-intensive career varies substantially by field, and trainees should approach certain specialties with a clear understanding of the additional negotiation and institutional support required.”

Additionally, the new Table 1 includes a “Potential Red Flags” column that gives trainees concrete warning signs to watch for when evaluating specialties and programs (e.g., departments primarily driven by clinical revenue with limited research infrastructure; absence of physician-scientists in leadership roles; inability to reduce clinical effort).

(3) Where will be the jobs for physician-scientists who have an MD ± PhD and want to do research and discovery? How many openings will there be for physician-scientists in academia 5–10 years from now? In industry? How are recent events in Washington affecting the continuation of those jobs?

after careful consideration, we believe that a detailed treatment of labor market projections, industry trends, and the effects of federal funding policy on the physician-scientist workforce falls outside the scope of this article, which is focused on the decision-making process for specialty selection. We note that the workforce question has been the subject of several recent analyses and commentaries (e.g., Milewicz et al., ASCI/AAP/APSA workforce reports) and feel that a thorough treatment would warrant a dedicated manuscript. We have not added this content but acknowledge the reviewer’s point in our thinking about future work.

(4) Should one of the “smart choices” in the article’s title be where you do the residency, and not just which residency you do? How important is it to be at a successful, research-intensive medical center/university, both during and after residency and fellowship training? If being in an institution where there are numerous very successful physician-scientists and scientists improves the likelihood of being able to sustain a physician-scientist career, how should graduating students improve their chances of being at one of those institutions?

This is an excellent point, and we agree that institutional environment is at least as important as specialty choice itself. We have made several changes to address this.

In the Introduction, we have added the statement: “Importantly, the ability to sustain a physician-scientist career is often determined as much by the institutional environment and training program as by the specialty itself.” This signals early in the manuscript that “where” is as critical as “which.”

In the new Table 1, we have included a row on “Institutional Environment and Culture” as one of the five key decision factors, with the explicit note that institutional commitment is often more determinative than specialty alone in enabling long-term success as a physician-scientist.

We have also added a dedicated paragraph advising trainees to assess the broader institutional environment by examining: (i) the number of R01-funded investigators within the department, (ii) the presence of institutional training grants (e.g., T32 programs), and (iii) the track record of trainees transitioning from mentored (K) awards to independent (R) funding. We direct trainees to publicly available resources such as NIH RePORTER and the Blue Ridge Institute for Medical Research rankings.

Finally, we have added a concluding sentence to the protected time section: “Taken together, these factors reinforce that institutional environment and departmental culture are often as determinative as specialty choice itself in shaping a sustainable physician-scientist career.”

(5) In every clinical discipline, there are departments that value physician-scientists more than other departments and invest accordingly. What advice would the authors give to help graduating students identify those departments?

This point is closely related to Point #4, and we have addressed it through the same set of revisions. The new paragraph on evaluating institutional environments provides concrete, actionable guidance for trainees on how to assess departmental commitment to physician-scientists, including specific metrics (R01 density, T32 presence, K-to-R transition rates) and publicly accessible tools (NIH RePORTER, Blue Ridge Institute rankings).

The new Table 1 “Potential Red Flags” column highlights warning signs that a department may not be supportive of physician-scientist careers, including: departments primarily driven by clinical revenue (RVUs) with limited research infrastructure; lack of protected time enforcement; minimal NIH funding; and absence of physician-scientists in leadership roles.

We have also expanded the existing discussion in the section on mentorship and residency selection, where we already noted the value of identifying departments with T32 grants and active physician-scientist mentors. The revised text now more explicitly connects these markers to the departmental evaluation process.

We believe these revisions substantially strengthen the manuscript and are grateful for the reviewers’ constructive feedback.
